# Limited positive effects on jump-landing technique in girls but not in boys after 8 weeks of injury prevention exercise training in youth football

**DOI:** 10.1007/s00167-019-05721-x

**Published:** 2019-09-20

**Authors:** Hanna Lindblom, Markus Waldén, Siw Carlfjord, Martin Hägglund

**Affiliations:** 1 Division of Physiotherapy, Department of Medical and Health Sciences, Linköping University, Linköping, Sweden; 2 Sport without Injury ProgrammE (SWIPE), Department of Medical and Health Sciences, Linköping University, Linköping, Sweden; 3 Division of Community Medicine, Department of Medical and Health Sciences, Linköping University, Linköping, Sweden; 4 Department of Orthopaedics, Hässleholm-Kristianstad Hospitals, Hässleholm, Sweden; 5 Football Research Group, Department of Medical and Health Sciences, Linköping University, Linköping, Sweden

**Keywords:** Neuromuscular training, Movement quality, Effect mechanisms

## Abstract

**Purpose:**

To evaluate changes in jump-landing technique in football-playing boys and girls after 8 weeks of injury prevention training.

**Methods:**

Four boys’ and four girls’ teams (mean age 14.1 ± 0.8 years) were instructed to use either the original *Knee Control* injury prevention exercise programme (IPEP) or a further developed IPEP, *Knee Control* + *,* at every training session for 8 weeks. Baseline and follow-up testing of jump-landing technique included drop vertical jumps (DVJ), assessed subjectively and with two-dimensional movement analysis, and tuck jump assessment (TJA).

**Results:**

Only minor differences in intervention effects were seen between the two IPEPs, and results are therefore presented for both intervention groups combined. At baseline 30% of the boys showed good knee control during the DVJ, normalised knee separation distances of 77–96% (versus hip) and a median of 3 flaws during the TJA. Among girls, 22% showed good knee control, normalised knee separation distances of 67–86% and a median of 4 flaws during the TJA. At follow-up, boys and girls performed significantly more jumps during TJA. No changes in jump-landing technique were seen in boys, whereas girls improved their knee flexion angle at initial contact in the DVJ (mean change + 4.7°, *p* < 0.001, 95% CI 2.36–6.99, *d* = 0.7) and their TJA total score (− 1 point, *p* = 0.045, *r* = − 0.4).

**Conclusion:**

The study showed small positive effects on jump-landing technique in girls, but not in boys, after 8 weeks of injury prevention training.

**Level of evidence:**

Level II.

**Trial registration:**

Clinical Trials gov identifier: NCT03251404

**Electronic supplementary material:**

The online version of this article (10.1007/s00167-019-05721-x) contains supplementary material, which is available to authorized users.

## Introduction

Anterior cruciate ligament (ACL) injury is a severe injury that has both short-term consequences, with long lay-off from sports and secondary injuries, and long-term consequences, such as early onset osteoarthritis [10, 12]. Additionally, a meta-analysis showed that while 81% of athletes returned to sport after surgery, only 65% returned to their pre-injury level of sports, and 55% returned to competitive sport [1]. Video analyses of injury situations in male and female team sports have shown that ACL injuries frequently occur in association with valgus collapse and tibial rotation from a nearly extended knee position [17, 31, 41]. ACL injuries usually occur shortly after initial ground contact in a cutting manoeuvre or one-legged landing [17, 31, 41]. Hence, it has been suggested that injury prevention exercise programmes (IPEP) should incorporate cutting and landing technique drills where excessive knee valgus is avoided and adequate knee flexion angles are attained [17].

A number of IPEPs, such as *Knee Control*, the 11 + and 11 + Kids programmes have been developed for use in the warm-up before football practice. These IPEPs have been efficacious in preventing injuries in both boys’ [35, 38] and girls’ football [38, 40]. The effect mechanisms by which the IPEPs reduce injury risk are, however, not fully understood [34, 37]. The programmes are believed to affect potential risk factors for ACL injury, [37] and change motion patterns and reduce neuromuscular deficits [34]. More research is, however, needed on the programmes that have the potential to reduce ACL injury risk [33]. Few studies have evaluated effect mechanisms of IPEPs in youth football, and these have investigated different IPEPs and different tests. No study has focused on the effect mechanisms of the *Knee Control* IPEP.

According to data from feasibility studies, fidelity with *Knee Control* is not optimal, with coaches modifying programme content and dosage, potentially limiting the preventive effect [21]. A qualitative study among coaches revealed that they wanted better programme fit and higher buy-in from the players [22]. This feedback and knowledge led the research group to develop the original *Knee Control* IPEP further into *Knee Control* + , which was also evaluated in this study.

The aim of the study was to evaluate changes in jump-landing technique in football-playing boys and girls after 8 weeks of injury prevention training.

## Materials and methods

The study was carried out during the second half of the competitive season in the autumn of 2017, between the middle of August when schools started, and end of October 2017 when the outdoor season ended. Jump-landing technique was evaluated during drop vertical jumps (DVJ) and tuck jump assessment (TJA) at baseline and at follow-up after approximately 8 weeks of injury prevention training.

### Participants

A convenience sample of four boys’ and four girls’ football teams were included, comprising 158 players aged 13–16 years. Teams with scheduled training sessions at least twice a week were considered eligible. The exclusion criterion was teams with previous experience of regularly using *Knee Control* or another IPEP in the preceding year.

For inclusion, the players had to be physically healthy and able to participate in testing with maximum effort. Background information about the players was collected from questionnaires at baseline.

### Interventions

Two different IPEP versions were introduced by randomisation in four teams each. Four teams were instructed to use the original *Knee Control* IPEP (Knäkontroll, SISU Idrottsböcker, 2005) with the added running warm-up that was introduced as a mobile application/webpage in 2012, and four teams used the *Knee Control* + . Both interventions were preceded by 5 min of standard warm-up running exercises with focus on knee control, followed by the same six principal neuromuscular preparation exercises or training aims for approximately 15 min: one-legged knee squats, pelvic lifts (hamstring strengthening), two-legged knee squats, the bench (core stability), lunges and jump/landing technique. The differences between the original *Knee Control* IPEP and *Knee Control* + were mainly the amount of possible progressions, with four different levels and an additional partner exercise in the original *Knee Control* IPEP, while *Knee Control* + had more options for progression with 6–10 different levels of increasing difficulty of exercises. Both IPEPs were led by the coach and, for practical reasons, all players in the team used the same level of the exercises, meaning that the exercises were progressed on a team-basis when the coach assessed that the players’ technique and neuromuscular control had improved.

The IPEP was implemented during an ordinary training session where all coaches received written and oral information from physiotherapists about the programme, and also practised the exercises. When possible, the players took part as well. The teams were recommended to use the programme at every training session (2–3 times per week) during the 8-week study period. Teams were recommended to use the 5-minute warm-up running exercises before matches as well. Teams were instructed to use all six IPEP exercises and start with the basic level, and then progress after 2 weeks of training. At this time the first author phoned the coaches to follow-up on the training and to discuss whether any exercises needed to be modified or replaced, and also encouraged the coaches to start progressing the training. Coaches were asked to report any adverse events throughout the intervention period.

### Testing procedures

Players from one team at a time were tested at baseline and follow-up. For all but one team, testing could be scheduled at the same time of the day at baseline and follow-up, whereas one of the boys’ teams was tested after lunch at baseline and in the evening at follow-up. Testing was done indoors in the same venue for all teams to standardise the test environment and ground conditions. Participating players were asked to refrain from physically exhausting training on the day before testing. Prior to testing all players took part in the 5-min running warm-up from the *Knee Control* IPEPs led by two physiotherapy students.

The full test battery included tests of agility, hop and sprint performance as well as jump-landing technique used in the following order: DVJ, agility *t*-test, single-leg hop for distance, 505 agility test, side-hop test, 10 and 20 m sprint test, TJA and countermovement jump test. Only the results of the DVJ and TJA will be presented in this manuscript. The testing order of the players was the same during all tests and it took about 2 h to complete the test battery for the whole team. To facilitate analysis all players were recommended to wear tight shorts, t-shirt, short socks and indoor shoes. Five players in one of the teams showed up without shoes and did the tests barefoot at both baseline and follow-up.

### Jump-landing technique assessments

Jump-landing technique was assessed by studying neuromuscular control in the DVJ and TJA. Both tests were filmed with two GoPro Hero5 cameras, one from the frontal and one from the lateral view. The cameras were synchronised with a GoPro smart remote control to start and stop filming at the same time. The films were scrutinised by the first author, a physiotherapist with 18 years’ experience as a group training instructor, using the Windows media player as many times as necessary to be certain about the judgment and in both real-time and slow-motion.

#### Drop vertical jump assessment

For the DVJ a test leader fitted all players with markers on the greater trochanters, the centre of the patellae, the lateral malleoli and the lateral epicondyle of the right femur at both test occasions. The player stood on a 30 cm high and 50 cm wide box with the feet 35 cm separated, dropped down from the box and immediately made a vertical jump to try to reach an overhead target positioned 2.6 m above. All players practised at least three times before performing three test trials.

The frontal plane knee control during the DVJ was assessed according to the criteria used by Nilstad et al. [27], i.e., knee alignment and/or presence of valgus and/or medio-lateral movement of one or two knees during the jump (with 0 representing good control, 1 reduced control and 2 poor control) (Additional file 1). The assessment focused on the first drop, the landing and the preparation for take-off. All three jumps for the same player were assessed and the film representing the worst technique was used for the analysis. The inter-rater agreement was substantial to almost perfect (70–95% agreement and kappa values *κ* = 0.52–0.92) when classifying female elite football players [27]. The test has also been shown to identify individuals with high knee valgus angles [27].

Objective 2D motion analysis of the jump-landing technique was made by measuring hip, knee and ankle separation distances from a frontal view [28, 29] and by measuring knee flexion angle from the sagittal view [4] using Dartfish software (Dartfish Pro Suite 7) (Additional file 2). The exact distance between the hip markers was used to calculate the normalised distance between the knee markings and ankle markings [28] in the following three time points: T1 (*initial contact*, the frame where the player’s feet just touched the ground), T2 (*maximum knee flexion*) and T3 (*preparation for take-off*, representing the frame where the player displayed the worst neuromuscular control between T2 and take-off from the ground). The knee flexion angle was measured at T1 and T2 from the lateral view. The validity of the test has been studied in female youth football players showing that low normalised knee separation distances were associated with increased lower extremity injury risk and knee injury risk [30]. High test–retest reliability of the hip separation distances (intraclass correlation coefficients = 0.94–0.96) has been shown in female athletes [28].

#### Tuck jump assessment

TJA was used to measure jump-landing technique subjectively using ten different criteria [26] (Additional file 3). The dichotomised grading scale proposed by Herrington et al., [13] was used. The player jumped repeatedly for 10 s and attempted to lift the knees to hip level (parallel to the ground) during the jump and start a new jump immediately upon landing. Free practice was allowed before the single test trial. Both high inter-rater reliability (80–100% agreement and kappa value *κ* = 0.88) and intra-rater reliability (87–100% agreement and kappa values *κ* = 0.86–1.00) were reported across the ten scoring criteria in male and female athletes [13].

### Ethics approval and consent to participate

The study was approved by the regional ethical review board in Linköping, Sweden: Dnr 2017/294-31. Players and their legal guardians received written information about the study and gave written informed consent before study commencement. Players depicted in the additional files specifically consented to their pictures being shown in research presentations.

### Statistical analysis

A sample size calculation was done for the analyses of performance effects of the *Knee Control* IPEPs (which is presented elsewhere) and not specifically for the analyses of jump-landing technique presented in the current paper.

Due to floor and ceiling effects for the subjective assessments of DVJ and TJA (no room for improvement in players with good technique at baseline and no room for deterioration in players with poor technique) these results were primarily presented as a distribution of test scores in the boys’ and girls’ teams and as change of normalised knee separation distances at T1, T2 and T3. In addition, baseline and follow-up comparisons of boys and girls were made using paired samples *t* test for the 2D motion analysis in Dartfish and Wilcoxon signed-ranks test for the subjective assessments. Analyses were made according to the intention-to-treat principle.

For the within-group comparisons effect sizes, Cohen’s *d* was calculated for parametric data using mean values and standard deviations, and *r* was calculated for non-parametric data based on *Z*/$\sqrt{N}$, where *N* equals the number of observations. Effect sizes were interpreted as: small *d* = 0.2, medium *d* = 0.5, and large *d* = 0.8 or small *r* = 0.1, medium *r* = 0.3 and large *r* = 0.5 [6].

## Results

At baseline 115 players (66 boys, 49 girls) participated and at follow-up 77 players returned, of whom 74 (47 boys, 27 girls) were analysed. Three players were excluded from analyses due to injuries preventing full participation at follow-up. Player demographics can be found in Table 1. No adverse events were reported during the intervention period. The number of training sessions with the *Knee Control* IPEPs varied between teams (training dose boys’ teams: 11, 12, 14 and 21 sessions, girls’ teams: 11, 11, 11 and 18 sessions) and also the time spent on the programme (10–30 min). In the objective 2D motion analysis of DVJ the markers were not visible at all time-points for one player and an additional seven videos had markers that were obstructed in some time points. For one boy no frontal view video was obtained during the TJA due to technical error at baseline and this test was excluded.

**Table 1:** Demographics of included players

	Boys (n = 47)	Girls (n = 27)
*Knee control*	21	8
*Knee control* +	26	19
Age, years	14.2 ± 0.7	14.0 ± 0.9
Body mass index at baseline, kg/m^2^	19.4 ± 2.2	19.6 ± 2.4
Menarche yes	–	19
Years of football experience	7.5 ± 2.3	7.0 ± 2.0
Active in other sports	One other (17); two other (6)	One other (11); two other (2)
Football profile at school	22	8
Other sports profile at school	8	3
Football training sessions/week at baseline	4.5 ± 1.2	4.4 ± 1.3
Perceived training volume^a^ at baseline	5.9 ± 1.0	5.4 ± 0.8
Previous experience of using the *Knee control* IPEP	Yes, regularly (0); yes, sporadically (12); no (34)	Yes, regularly (3); yes, sporadically (17); no (7)

Values are *n*, or mean ± standard deviation unless otherwise stated

*SD* standard deviation

^a^Likert scale 1–7, where 1 represents extremely low training volume and 7 extremely high training volume

When first analysing the results separately for each intervention group, irrespective of sex, no changes were seen in the performance of DVJ over time except for an increase in knee flexion angle at initial contact (+ 3.4°, 95% CI 0.8–6.0, *p* = 0.013, *d* = 0.4) in the *Knee Control* + group. No change in TJA was seen except for an increased number of jumps at follow-up in both groups. As only minimal differences were seen between the *Knee Control* IPEPs, further analyses were hereafter made with both intervention groups combined.

**Fig. 1:**
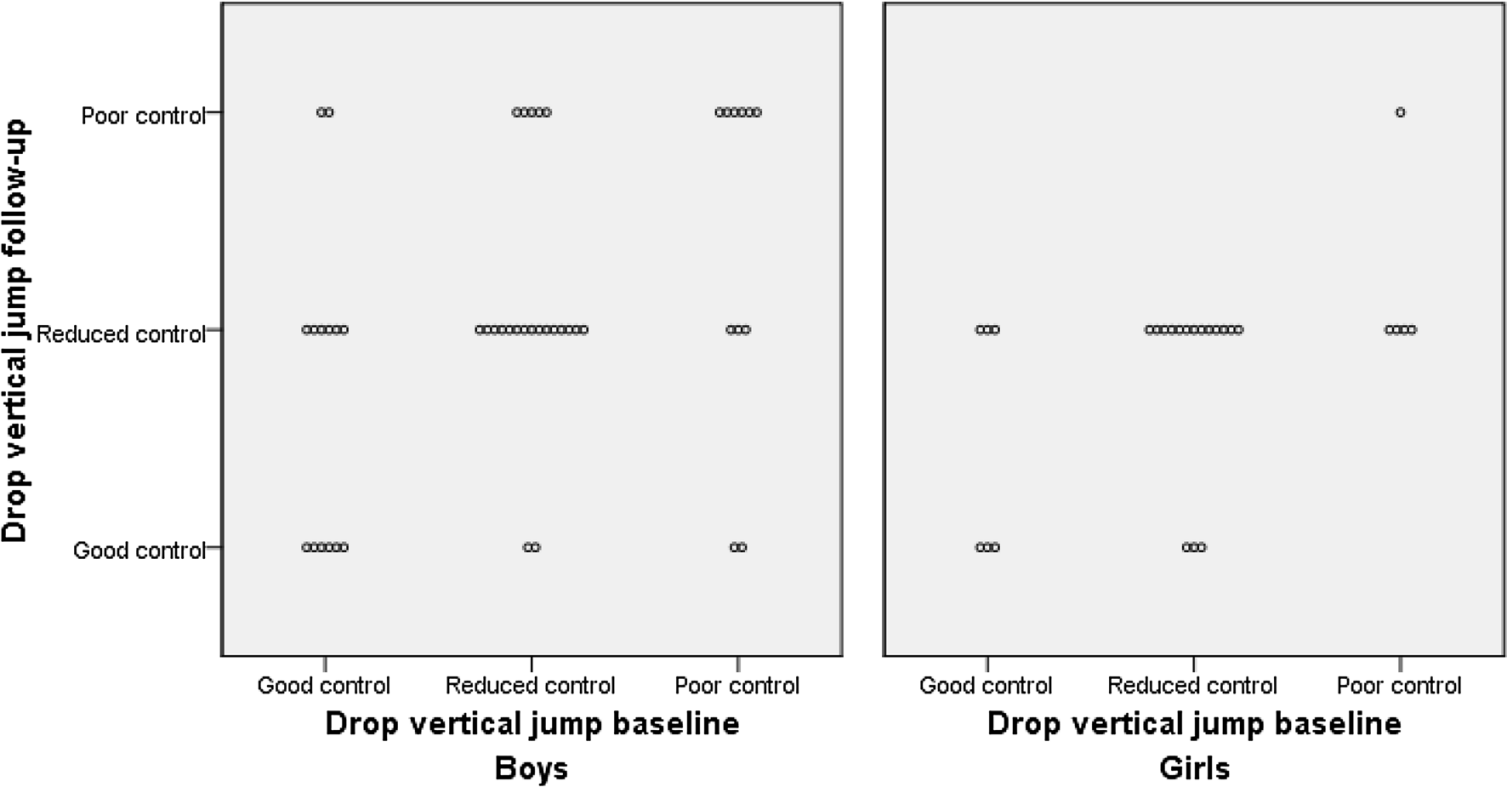
Distribution of subjective assessment of the drop vertical jump visually shown at baseline and follow-up, where each circle represents an individual

In the baseline subjective assessment of the DVJ, 30% of the boys and 22% of the girls displayed good knee control (Table 2). There was no change in the performance of the DVJ in either boys or girls over time in the subjective assessment (Fig. 1). There was a small but significant change in normalised knee separation distance at T1 in the boys with worse performance at follow-up (mean change − 4%, *p* = 0.042, 95% CI − 8.4 to − 0.2, *d* = -0.30). In girls a significantly higher knee flexion angle at initial contact (T1) was seen at follow-up (mean change + 4.7°, *p* < 0.001, 95% CI 2.4–7.0, *d* = 0.7).

**Table 2:** Subjective assessment and results of the objective 2D motion analysis of the drop vertical jump in boys and girls at baseline and follow-up

	Boys (n = 47)		Girls (n = 27)	
	Baseline	Follow-up	Baseline	Follow-up
*DVJ subjective assessment*				
Good control n (%)	14 (30%)	10 (21%)	6 (22%)	6 (22%)
Reduced control n (%)	22 (47%)	24 (51%)	16 (59%)	20 (74%)
Poor control n (%)	11 (23%)	13 (28%)	5 (19%)	1 (4%)
*2D analysis of DVJ*^a^				
NKSD T1% (SD)	96 ± 15	92 ± 14*	86 ± 9	86 ± 12
NKSD T2% (SD)	84 ± 27	80 ± 26	76 ± 16	76 ± 20
NKSD T3% (SD)	77 ± 25	76 ± 25	67 ± 21	71 ± 14
Knee flexion angle T1 degrees (SD)	26.9 ± 7.1	29.0 ± 8.2	22.7 ± 6.0	27.4 ± 5.0*
Knee flexion angle T2 degrees (SD)	93.1 ± 14.8	90.6 ± 11.6	87.0 ± 10.3	89.5 ± 10.5

Values are n (percent), ratio ± standard deviation or degrees ± standard deviation

*DVJ* drop vertical jump, *NKSD* normalised knee separation distance, *SD* standard deviation. The table displays *NKSD* at T1: initial contact, T2: maximum knee flexion and T3: preparation for take-off

^*^Indicates significantly different results (*p* < 0.05) compared to baseline

^a^*n* = 44–46 boys and 25–27 girls due to markers being obstructed during some time points of the video assessment

**Fig. 2:**
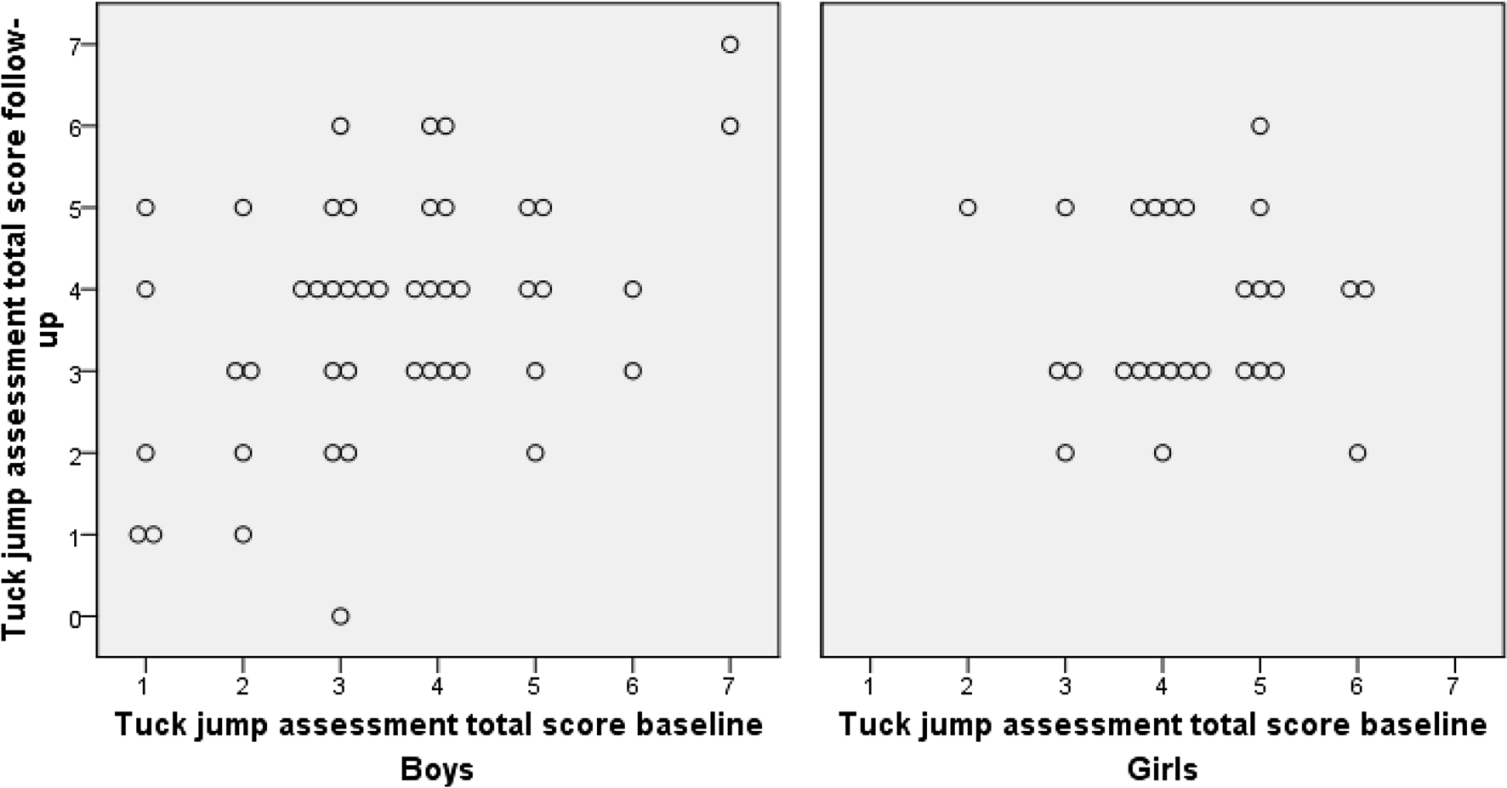
Tuck jump assessment total score for boys and girls separately, visually shown at baseline and follow-up, where each circle represents an individual

TJA scores are shown in Table 3. No change was seen in the TJA total score in boys, while an improvement was seen in girls over time with the median score changing from 4 at baseline to 3 at follow-up (*p* = 0.045, *r* = − 0.4) (Fig. 2). Both sexes performed significantly more jumps at follow-up compared to baseline. When analysing the results according to each criterion no differences were seen between baseline and follow-up in boys for any criteria, whereas there was a significant improvement in girls for two criteria.

**Table 3:** Number of players presenting with each flaw during the tuck jump assessment

Tuck jump criteria	Boys (n = 46)^a^		Girls (n = 27)	
	Baseline	Follow-up	Baseline	Follow-up
1. Lower extremity valgus at landing	20 (43%)	26 (55%)	19 (70%)	20 (74%)
2. Thighs do not reach parallel (peak of jump)	28 (60%)	31 (66%)	20 (74%)	19 (70%)
3. Thighs not equal side-to-side (during flight)	24 (51%)	26 (55%)	9 (33%)	11 (41%)
4. Foot placement not shoulder width apart	29 (62%)	29 (62%)	13 (48%)	11 (41%)
5. Foot placement not parallel (front to back)	12 (26%)	10 (21%)	5 (19%)	8 (30%)
6. Foot contact timing not equal	3 (6%)	3 (6%)	0 (0%)	1 (4%)
7. Excessive landing contact noise	9 (19%)	12 (26%)	16 (59%)	10 (37%)
8. Pause between jumps	3 (6%)	3 (6%)	7 (26%)	2 (7%)*
9. Technique decline prior to 10 s	4 (9%)	5 (11%)	8 (30%)	1 (4%)*
10. Does not land in same footprint	29 (62%)	30 (64%)	19 (70%)	17 (63%)
Tuck jump assessment total score median (IQR)	3 (1)	4 (2)	4 (1)	3 (2)*
Number of jumps mean ± SD	15.5 ± 1.7	16.4 ± 2.0*	15.1 ± 1.7	16.0 ± 1.1*

Values are n (percent) or mean ± standard deviation

*IQR* interquartile range, *SD* standard deviation

*Indicates significantly different results (*p* < 0.05) compared to baseline

^a^One player was missing from the assessment due to technical error when filming

## Discussion

The main finding of the study was that small improvements, representing medium to large effect sizes, were observed in girls, irrespective of intervention, for knee flexion angle, TJA total score, two of the TJA criteria and number of jumps during the 10 s of TJA. No effect was seen in boys except an increase in the number of jumps during the 10 s of TJA. Differences in effects between the two different interventions were minor.

### Tuck jump assessment

The small improvements seen in TJA among the girls are positive, as girls have a higher risk of acute knee injury than boys [39]. The potential for improvement may also have been higher in girls as they performed worse at baseline. According to Myer et al. [25] individuals presenting ≥ 6 flaws in the TJA should be specifically targeted for preventive actions. The players in the current study showed a median of three to four flaws, i.e., they performed rather well already at baseline and had less room for improvement, but a significant improvement in the girls was found nonetheless. Another study also found improvement in the TJA after a neuromuscular training intervention, but similar improvement was observed also in the control group [16]. In future studies it is possible that using the modified TJA scoring with a three-graded scale [8], instead of the dichotomous scale used in the current study, might capture smaller changes in neuromuscular control as a result of preventive training.

A critique of the TJA is that it may not measure fatigue in a structured way, as the speed of jumping is not controlled for and one way for athletes to improve the total score may be to jump at a slower rate [36]. In the current study, the improvement seen in the TJA total score in the girls occurred in combination with an increased number of jumps. This strengthens the results on the TJA total score, as an increase in jump rate is more demanding for the player and may challenge the neuromuscular control more. This increase in jump rate may also be interpreted as a training effect of the *Knee Control* IPEPs suggesting increased strength, power and plyometric performance.

### Drop vertical jump

In the DVJ no effect was shown on landing biomechanics, except for an increase in knee flexion angle at initial contact in girls. The results are in line with two other studies in girls’ football that also reported no effect from 2D or 3D motion analyses of DVJ from preventive training using the WIPP (Warm-up for Injury Prevention and Performance) programme [11] and the 11 + [2]. The results do, however, diverge from Lim et al., [20] who showed improvements in both knee flexion angles and knee separation distance during rebound jumps after using a modified version of the PEP (Prevent injury and Enhance Performance) programme in youth female basketball for 8 weeks. In future studies, inclusion of electromyographic activity measures may be valuable to understand better the effect mechanisms of the IPEPs. As neither boys nor girls in the current study presented with distinct valgus alignment (normalised knee separation distances < 60%) according to the criteria by Noyes et al., [29] the room for improvement in the DVJ may have been limited, explaining the absent effect.

### Effects on potential neuromuscular injury risk factors

Knee valgus seemed to be unaffected by the interventions as assessed in the TJA criteria of lower extremity valgus as well as in the subjective and objective assessments of the DVJ. Movement patterns that are coupled to high knee valgus moment and valgus angles have been shown to be associated with an increased risk of ACL injury [14], although other subsequent studies have not confirmed these results [18, 19]. While some argue that knee valgus is a natural movement, that needs to be controlled rather than avoided [7], decreasing lower extremity valgus and increasing knee flexion angles in jumping/landing and cutting are often seen as important targets for IPEPs [17, 19] and in the current study the girls’ knee flexion angles improved. It should be noted that even though the effect size was large, the minimal clinically relevant change in knee flexion angle is not known. Additionally, an overall better jump-landing technique at follow-up was seen among the girls according to the TJA total score. How this affects the overall risk of injury is not known, as few of the individual TJA criteria showed significant improvements. Considering that the risk of acute knee injury is higher in girls than in boys, the study shows positive results by indicating that movement patterns may be changed by the interventions.

### Methodological considerations

Two-dimensional tests cannot assess rotation, such as internal and external rotation of the lower extremity but have been shown to have high reliability and adequate validity compared with 3D motion analysis [32]. However, due to the imprecision of the subjective assessments we saw considerable ceiling and floor effects, resulting in less room for improvement or decline in performance. One strength was that the results of the subjective DVJ assessment and the objective 2D motion analysis using normalised knee separation distances corroborated each other as no change in either test was seen. Another strength was the use of two different tests of jump-landing technique assessed in the same population making it possible to study the consistency of the results across assessments. Utilisation fidelity (if the programme was used as intended regarding exercise selection, dosage and progression) or exercise fidelity (whether the exercises were done with the correct technique) was not monitored as part of the study, which is a possible limitation, especially as the exercise fidelity with the *Knee Control* IPEP has been shown to be sub-optimal [23]. The potential for an IPEP to improve jump-landing technique may be influenced by, for instance, coaches’ instructions on exercise technique. Therefore, the coach may play a vital part in any observed effect or lack of effect on jump-landing technique.

Some other limitations include a shorter study duration than intended, due to the short time period between school starting after the summer break and the end of the football season. However, regarding neuromuscular control, a single exercise session with adequate feedback may be enough to affect jump-landing technique [5], even though this of course may not have long-lasting effects. Other limitations were the lack of blinding, as the analyst for practical reasons could not be blinded. Furthermore, other limitations were the lack of a pure control group, limited time for progression of the training and large drop out, especially among the girls, which limits the statistical power. When interpreting these results, one must bear in mind that the population was young, with rather large intra-individual differences within the same session. Additionally, the tests were unfamiliar to most and they had no established movement pattern when doing DVJs and TJAs at baseline, and hence, there is a possibility of a learning effect for the follow-up measurements. Additionally, results may be obscured by a maturing study population potentially changing their movement technique spontaneously. As in similar studies, we cannot be certain that changes seen in the tests will be transferred to real-life potentially harmful situations.

The results were not analysed based on the players’ performance at baseline even though individuals at high risk of ACL injury are believed to respond more favourably to IPEPs from a neuromuscular and biomechanical point of view [9, 24]. To make all players benefit more from IPEPs it has been suggested that training should be tailored towards the observed movement deficits [9, 15], which may be valuable in future studies. The feasibility of individualised tailoring of IPEPs in the real-world may, however, be questioned as this requires more experience and time from the coach.

This study extends understanding of the effect mechanisms of IPEPs, which is important from, for example, an implementation point of view. Earlier studies have shown that coaches modify the IPEPs, with unknown effects on prevention efficacy. As understanding of the effect mechanisms grows it may be possible to tailor programmes to fit the teams and, as a result, support successful adoption and maintenance of the IPEPs.

## Conclusion

This study showed small positive effects on knee flexion angle at initial contact after DVJ and in TJA total score after 8 weeks of IPEP training in girls. No changes between baseline and follow-up were seen in boys.

## Electronic supplementary material

Below is the link to the electronic supplementary material.

Supplementary file1 (DOCX 180 kb)

Supplementary file2 (DOCX 417 kb)

Supplementary file3 (DOCX 670 kb)
