# Supplementary material for: Limited positive effects on jump-landing technique in girls but not in boys after 8 weeks of injury prevention exercise training in youth football
Source: Knee Surg Sports Traumatol Arthrosc. 2019 Sep 20;28(2):528–37. doi: 10.1007/s00167-019-05721-x (PMC6994440; doi:10.1007/s00167-019-05721-x)
Supplement: Supplementary file 1 — Supplementary file1 (DOCX 180 kb) [file 167_2019_5721_MOESM1_ESM.docx]

**Examples of the subjective assessment of drop vertical jumps**


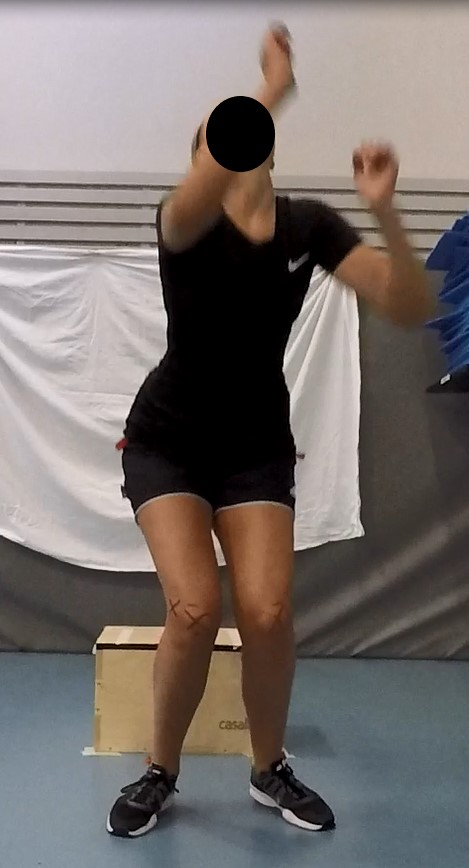

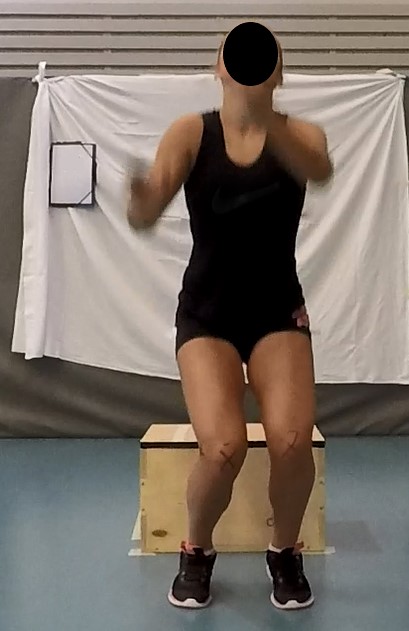
**
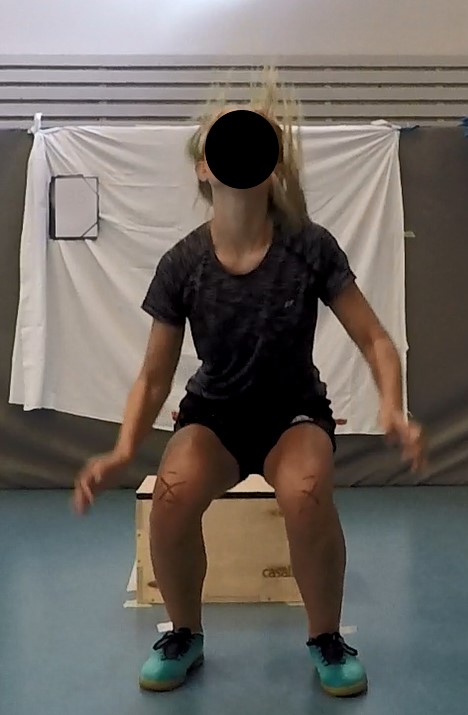
**

Reduced control

Poor control

Good control
