# Supplementary material for: Limited positive effects on jump-landing technique in girls but not in boys after 8 weeks of injury prevention exercise training in youth football
Source: Knee Surg Sports Traumatol Arthrosc. 2019 Sep 20;28(2):528–37. doi: 10.1007/s00167-019-05721-x (PMC6994440; doi:10.1007/s00167-019-05721-x)
Supplement: Supplementary file 2 — Supplementary file2 (DOCX 417 kb) [file 167_2019_5721_MOESM2_ESM.docx]

**Additional file 2**

**Examples of the 2D motion analysis of drop vertical jumps in Dartfish**

**
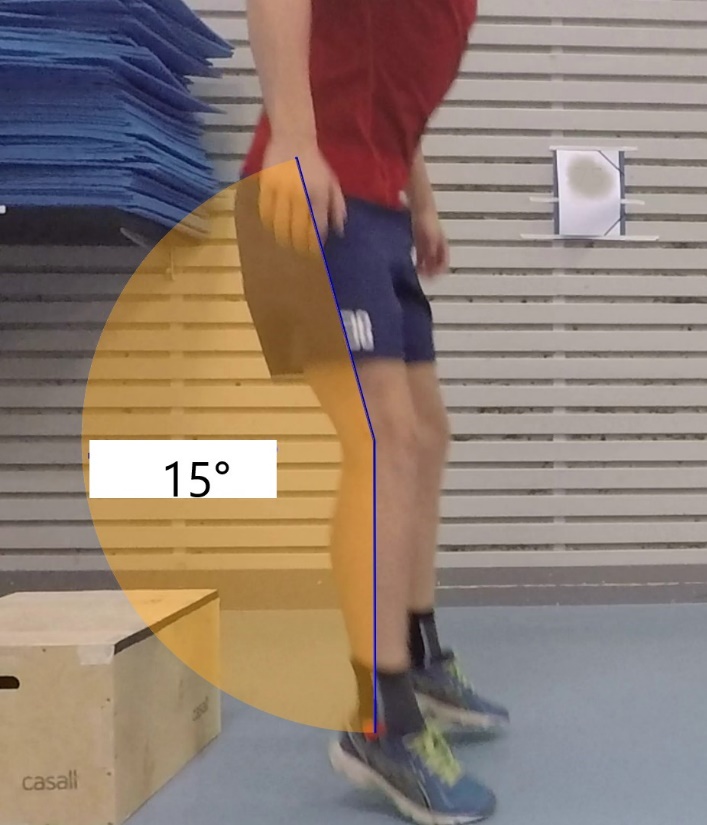
**
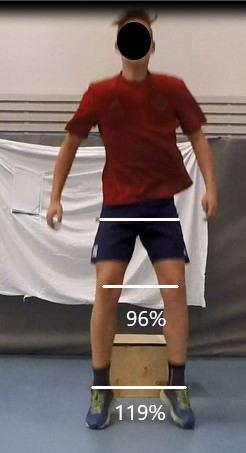


**T1 Initial contact; normalised knee and ankle separation distances, knee flexion angle**


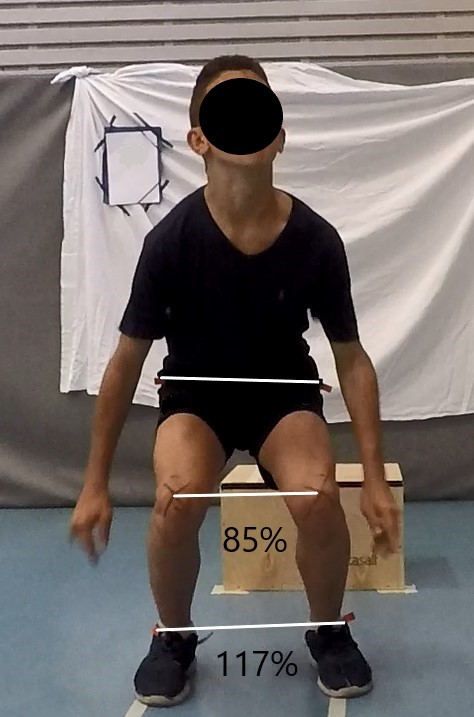

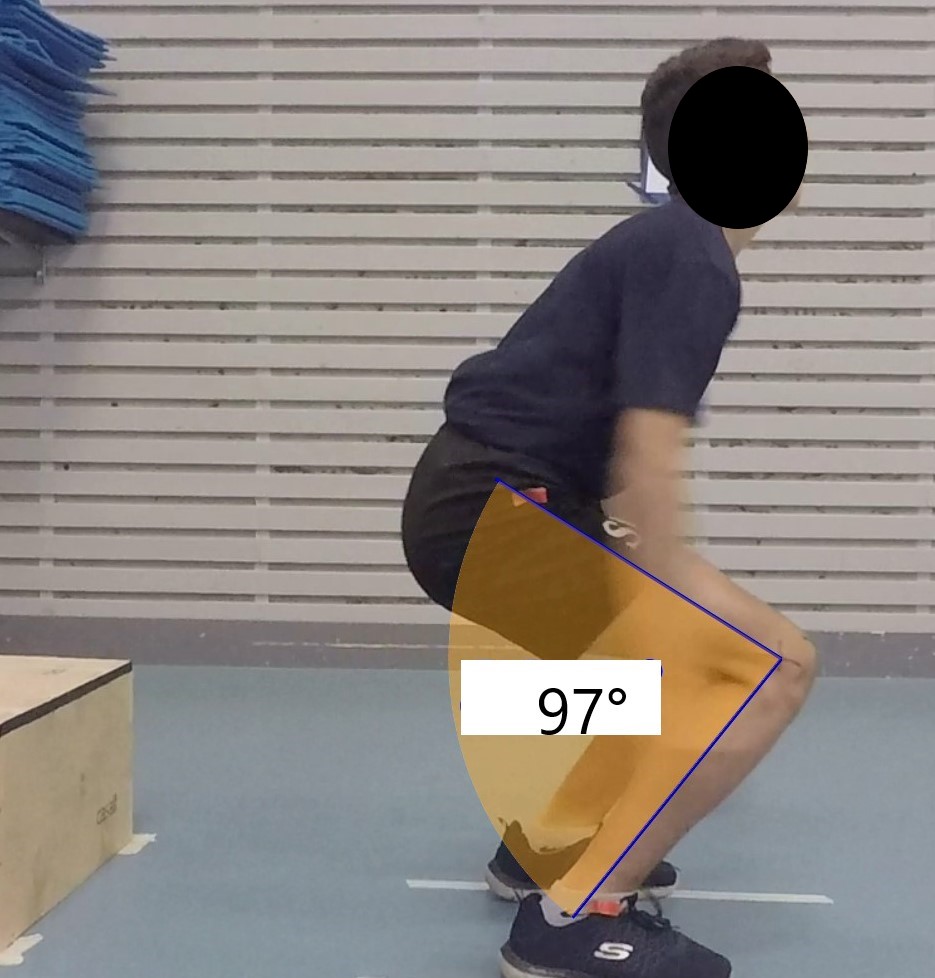


**T2 Maximum knee flexion; normalised knee and ankle separation distances, knee flexion angle**


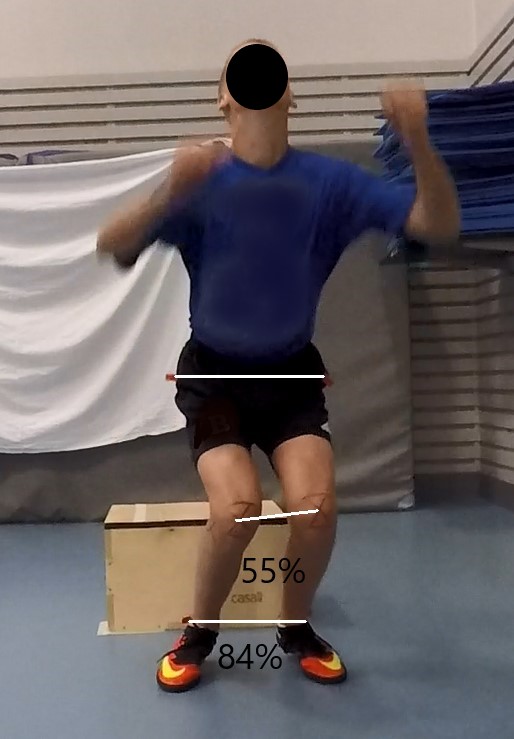


**T3 Preparation for take off; normalised knee and ankle separation distances**
