# Supplementary material for: Limited positive effects on jump-landing technique in girls but not in boys after 8 weeks of injury prevention exercise training in youth football
Source: Knee Surg Sports Traumatol Arthrosc. 2019 Sep 20;28(2):528–37. doi: 10.1007/s00167-019-05721-x (PMC6994440; doi:10.1007/s00167-019-05721-x)
Supplement: Supplementary file 3 — Supplementary file3 (DOCX 670 kb) [file 167_2019_5721_MOESM3_ESM.docx]

**Examples of the tuck jump assessment, criteria 1-10 representing different flaws**


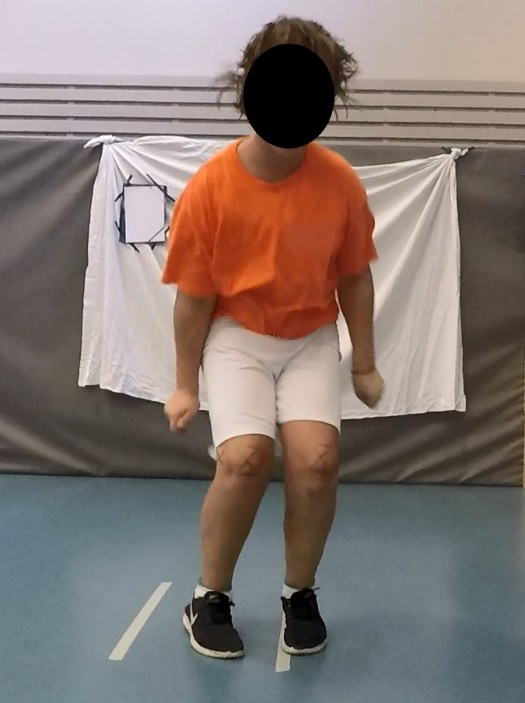

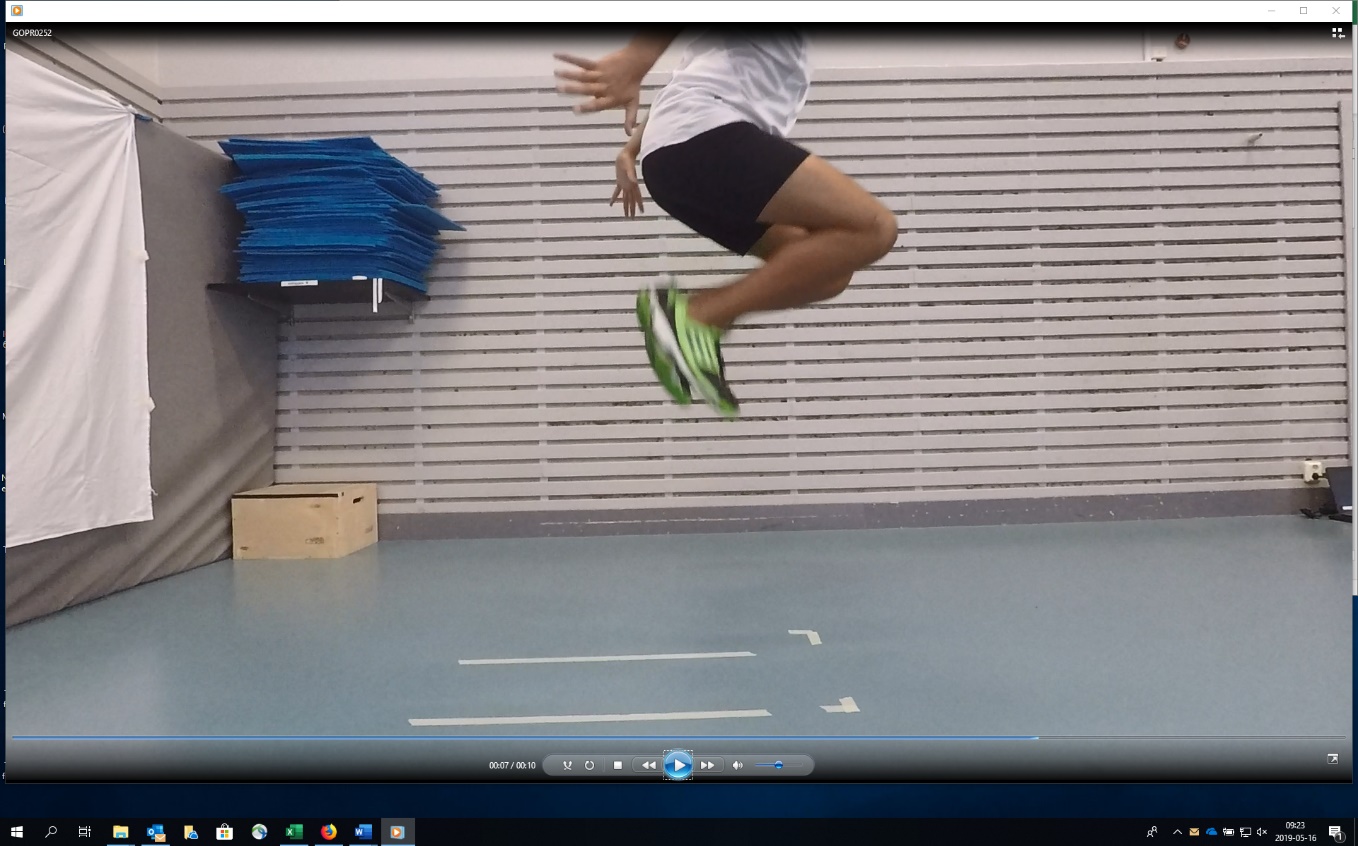


1. Lower extremity valgus at landing 2. Thighs do not reach parallel (peak of jump)


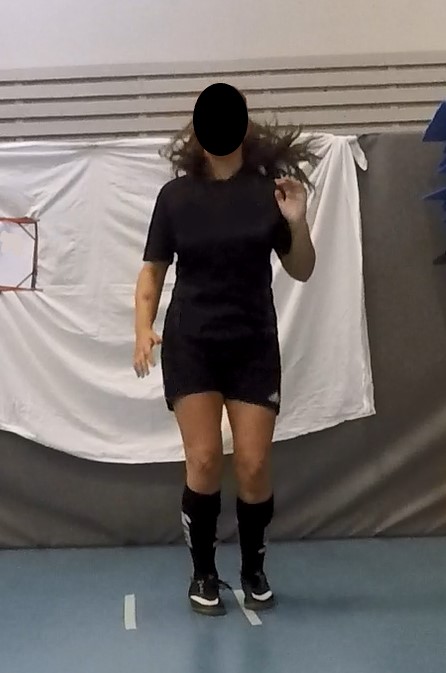

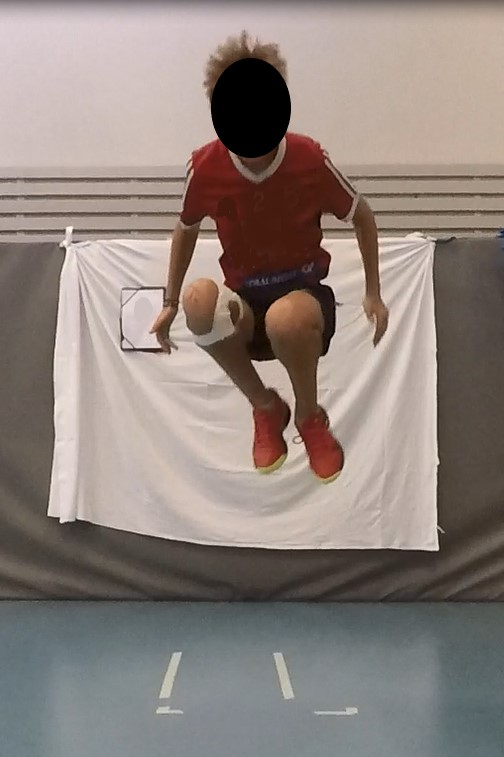


3. Thighs not equal side-to-side (during flight) 4. Foot placement not shoulder width apart


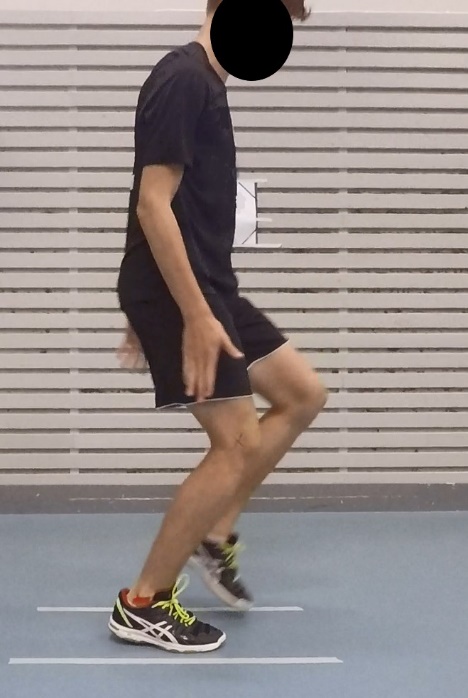

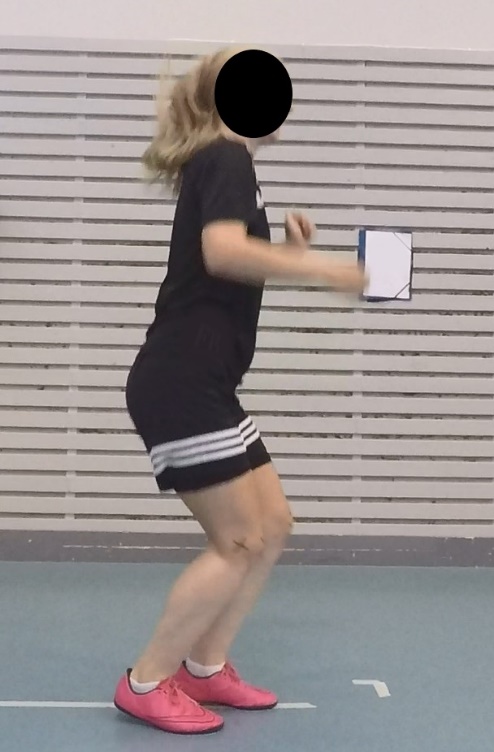


5. Foot placement not parallel (front to back) 6. Foot contact timing not equal

**Criteria not shown visually:**

7. Excessive landing contact noise

8. Pause between jumps

9. Technique declines prior to 10 seconds

10. Does not land in the same footprint (excessive in-flight motion)
